# Beamforming for Multi-Bit Intelligent Reflecting Surface with Phase Shift-Dependent Power Consumption Model

**DOI:** 10.3390/s24186136

**Published:** 2024-09-23

**Authors:** Huimin Zhang, Qiucen Wu, Yu Zhu

**Affiliations:** School of Information Science and Technology, Fudan University, Shanghai 200433, China; hmzhang21@m.fudan.edu.cn (H.Z.); qcwu21@m.fudan.edu.cn (Q.W.)

**Keywords:** beamforming optimization, generalized Benders decomposition, intelligent reflecting surface, phase shift-dependent power consumption

## Abstract

In recent years, the intelligent reflecting surface (IRS) has attracted increasing attention for its capability to intelligently reconfigure the wireless propagation channel. However, most existing works ignore the dynamic power consumption of IRS related to the phase shift configuration. This relationship gets even more intractable for a multi-bit IRS because of its nonlinearity and implicit form. In this paper, we investigate the beamforming optimization for multi-bit IRS-aided systems with the practical phase shift-dependent power consumption (PS-DPC) model, aiming at minimizing the power consumption of the system. To solve the implicit and nonlinear relationship, we introduce a selection matrix to explicitly represent the power consumption and the phase shift matrix of the IRS, respectively. Then, we propose a generalized Benders decomposition-based beamforming optimization algorithm in the single-user scenario. Furthermore, in the multi-user scenario, we design a coordinate descent-based algorithm and a genetic algorithm for the beamforming optimization. The simulation results show that the proposed algorithms significantly decrease the power consumption of the multi-bit IRS-aided systems.

## 1. Introduction

Recently, the rapid development of communication applications has placed higher demands on wireless communication, as reflected in the visions of the sixth generation, i.e., immersive communication, massive communication, hyper-reliable and low-latency communication, integrated sensing and communication, ubiquitous connectivity, and integrated artificial intelligence and communication [1]. On this basis, intelligent reflecting surfaces (IRSs) become a pivotal trend for advancing next-generation wireless communication systems. An IRS is a planar array composed of numerous controllable passive reflection elements [2,3,4]. An IRS reflects the incident signal toward the desired directions and achieves passive beamforming by independently controlling the phase shifts of these elements. Due to its passive characteristics, an IRS reconfigures the wireless propagation environment intelligently while having low power consumption. These capabilities of an IRS can significantly overcome the blockages, broaden the wireless coverage, and improve the quality of service (QoS) [5,6,7,8], thus allowing for IRSs to be employed in various communication scenarios, e.g., green communications [9], simultaneous wireless information and power transfer systems [10], unmanned aerial vehicle communications [11], integrated sensing and communication systems [12], the physical layer security of wireless networks [13], etc.

In the pursuit of efficient and green wireless communications for future networks, many studies have focused on energy efficiency and power consumption in IRS-aided systems. For example, the authors in [14] investigated and analyzed the effect of the IRS reflection elements’ quantity and the reflection resolution on network energy efficiency. In [15], the study researched the success probability and the energy efficiency in IRS-aided millimeter-wave networks, revealing the impact of the density of deployed IRSs. In [16], the authors performed a comprehensive analysis and comparison of the energy efficiency between IRS-aided systems and decode-and-forward (DF) relay systems, indicating that the IRS-aided systems perform better across various numbers of IRS elements and the mobile station positions. In [17], the authors jointly optimized the IRS coefficients, user transmit power, and linear receive filters to compare the active and passive IRSs concerning energy efficiency. In [18], the authors considered the IRS containing both active and passive reflection elements and optimized the number of these two types of elements to balance spectral efficiency and power consumption. In [19], for an IRS-aided system, the authors optimized the transmit power of the base station (BS), aiming to maximize energy efficiency with hardware impairments.

Furthermore, beamforming optimization concerning power consumption for IRS-aided systems is worth studying. On the one hand, beamforming is a signal processing technique to enhance the quality of the signal and has been widely used in various applications [20,21,22]. On the other hand, several previous works have investigated the green beamforming design for IRS-aided systems with various metrics. In [7], the authors considered energy efficiency as the optimization objective, in which the power consumption consists of the BS, IRS, and users. In [23], the authors proposed a green beamforming problem for an IRS-aided system, incorporating both spectral and energy efficiency in the objective function to ensure a trade-off between the power consumption and the achievable rate. The authors in [24] proposed a strategy based on a statistical channel model to maximize energy efficiency by optimizing the phase shift matrix and power allocation of the IRS, thereby reducing the optimization overhead of variables in each channel coherence time. The authors in [25] investigated a trade-off between minimizing power consumption and maximizing spectral efficiency while satisfying the QoS requirement, proposing a joint optimization scheme based on fractional programming. In [26], a resource allocation problem was formulated, in which the authors optimized the beamforming design to reduce the transmit power consumption. In addition, several other studies have studied beamforming optimization concerning power consumption or energy efficiency across various IRS-aided scenarios, e.g., wireless powered networks [27,28], internet of things networks [29,30], full-duplex systems [31], integrated communication, sensing and power transfer systems [32], secure integrated terrestrial–aerial networks [33], anti-jamming and secure transmission [34], satellite terrestrial relay networks [35], etc.

However, most existing works assume that the power consumption of the IRS is constant or solely dependent on the number of elements. Actually, these works have not considered the fact that for an element of the positive-intrinsic-negative (PIN) diode-based IRS, different phase shifts are associated with varying levels of power consumption, which has been evidenced in recent experimental results [36,37,38]. Specifically, the phase shifts of the PIN diode-based IRSs are controlled by changing the on/off-state of PIN diodes, where a PIN diode consumes $P_{\mathrm{PIN}}$ in the on-state, while it is power-free in the off-state [38]. For an IRS with numerous reflection elements, its dynamic power consumption can be rather high, constituting a significant proportion in the system, e.g., considering a 2-bit IRS equipped with 1600 elements, it is $P_{\mathrm{PIN}}=12.6\;\mathrm{mW}$ [38] and the dynamic power consumption is about 20 W. Therefore, an inaccurate power consumption model introduces additional errors and results in high power consumption in practical systems. The phase shift-dependent power consumption (PS-DPC) of IRSs should be considered in the beamforming optimization problem to minimize the system power consumption. Using the PS-DPC model, the power consumption should be allocated between the BS and IRS appropriately rather than taking a constant power consumption model for the IRS.

In our previous work [39], we minimized the system power consumption using the PS-DPC model for an IRS, considering the IRS phase shifts with a 1-bit resolution. In [40,41], the authors also considered a similar PS-DPC model for a 1-bit IRS and aimed to maximize energy efficiency. Nevertheless, these studies adopted a practical power consumption model of the IRS but only considered the 1-bit IRS, that is, each IRS element has two possible states and two different phase shifts. For the multi-bit IRS, each IRS element has four or more phase shifts, and the relationship between the dynamic power consumption and the phase shifts of the IRS becomes even more intractable due to its nonlinearity and implicit form. Therefore, the algorithms in these studies are only suited for a 1-bit IRS and can not be directly applied to a multi-bit IRS. In fact, as the IRS with a multi-bit phase shift resolution can lead to more precise beamforming, it is important and necessary to study this issue. To the best of our knowledge, there is no existing work on multi-bit IRS beamforming optimization with the consideration of the PS-DPC. Hence, we are committed to considering the PS-DPC model for the multi-bit IRS to design the beamforming optimization.

In this article, we investigate the beamforming optimization for multi-bit IRS-aided systems aiming at minimizing the total system power consumption of the BS and IRS for both single-user and multi-user scenarios. The main contributions are summarized as follows:

- In the single-user scenario, we consider a practical PS-DPC model and formulate a multi-bit IRS beamforming optimization problem to minimize the total system power consumption. The proposed PS-DPC model for the multi-bit IRS is better suited to the actual IRS-aided systems, in which the problem formulation and beamforming optimization are more challenging than the 1-bit IRS scenario. To deal with the difficulty of the implicit and nonlinear relationship between the PS-DPC and the phase shifts, we introduce a selection matrix to explicitly represent the PS-DPC and the phase shift matrix, respectively, and then propose a generalized Benders decomposition (GBD)-based algorithm to solve this mixed-integer nonlinear programming (MINLP) problem.
- We generalize the multi-bit IRS beamforming optimization problem to the multi-user scenario. We also solve the intractable implicit and nonlinear issue by explicitly representing the PS-DPC and the phase shift matrix, respectively. Then, we propose a coordinate descent (CD)-based algorithm and a genetic algorithm (GA)-based scheme to optimize the beamforming matrix of the BS and IRS, respectively. The CD algorithm has better performance, while the genetic algorithm has lower computational complexity.
- We present a range of simulation results that illustrate the effectiveness of our algorithms. Furthermore, we analyze the characteristics and performance of these simulation results. By effectively considering the PS-DPC, the simulation results show that the proposed algorithms achieve a balance of the power consumption of the BS and IRS, thus significantly reducing the total system power consumption compared to the baseline algorithms.

The rest of this paper is organized as follows. In Section 2, we introduce the system model, the power model, and the problem formulation. In Section 3, we solve the intractable nonlinearity to represent the power consumption of the IRS and present a GBD-based algorithm for the single-user scenario. Furthermore, we propose a CD-based algorithm and a GA-based scheme for the multi-user scenario in Section 4, where the representation of the power consumption is treated similarly to the previous section. In Section 5, we show various numerical results and the corresponding discussions. Finally, we conclude the paper in Section 6.

Notations: In this paper, $j=\sqrt{-1}$ denotes the imaginary unit. Regular letters, bold lowercase, and bold uppercase denote scalars, column vectors, and matrices, respectively. ${\left[\cdot \right]}_{m}$ denotes the *m*-th element of a vector, and ${\left[\cdot \right]}_{m,n}$ denotes the (m,n)-th element of a matrix. ${\left(\cdot \right)}^{*}$, ${\left(\cdot \right)}^{T}$, ${\left(\cdot \right)}^{H}$, and ${\left(\cdot \right)}^{-1}$ denote the conjugate, transpose, conjugate transpose, and inverse operators, respectively. Tr(·) denotes the trace of a matrix. E(·) denotes the expectation of a matrix. Arg(·) denotes the argument of a complex number. diag(·) denotes a diagonal matrix with the elements of a vector on its main diagonal. $I_{N}$ denotes an N×N identity matrix, 1 denotes an all-1 vector or an all-1 matrix, and 0 denotes an all-0 vector or an all-0 matrix. $C^{M\times N}$ represents the set of all M×N complex-valued matrices. Finally, CN(0,x) denotes the circularly symmetric complex Gaussian distribution with zero mean and covariance *x*.

## 2. System Model and Problem Formulation

### 2.1. System Model

**Figure 1:**
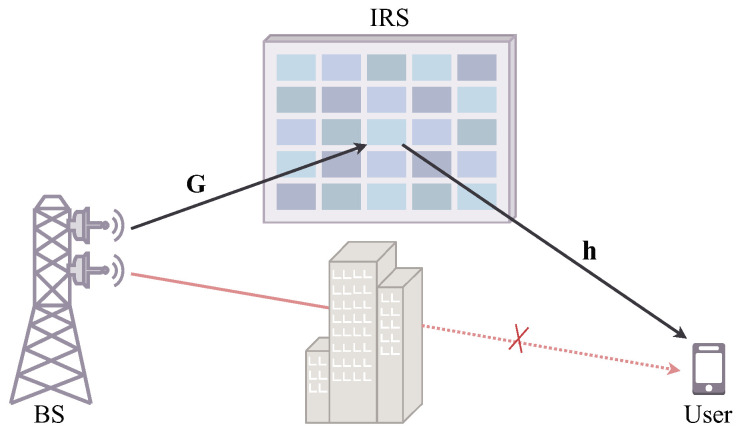
The system model for the downlink multi-bit IRS-aided system.

As shown in Figure 1, we consider a downlink narrowband system with a BS employing *N* transmit antennas, an IRS consisting of *M* reflection elements, and a single-antenna user. The BS is a uniform linear array and the IRS is a uniform planar array. The direct path between the BS and the user is assumed to be blocked. Therefore, the received signal at the user can be expressed as

(1) $$y=h^{H}\Phi Gfs+n,$$

where s∈C denotes the original symbol with $E\left\{s^{2}\right\}=1$, and $f\in C^{N\times 1}$ denotes the digital beamforming vector at the BS. The BS-IRS channel and the IRS-user channel, i.e., $G\in C^{M\times N}$ and $h\in C^{M\times 1}$, are both modeled as Rician channels. $\Phi =\mathrm{diag}\left(e^{j\theta_{1}},e^{j\theta_{2}},\ldots ,e^{j\theta_{M}}\right)\in C^{M\times M}$ denotes the diagonal phase shift matrix of the IRS. Specifically, the phase shift resolution of the IRS reflection elements is assumed to be *b* bits, which means the phase shift of the *m*-th element $\theta_{m}$ admits $L=2^{b}$ values, where $m\in M=\left\{1,2,\ldots ,M\right\}$, i.e., $\theta_{m}\in \left\{0,\Delta \theta ,\ldots ,\left(L-1\right)\Delta \theta \right\}$, where $\Delta \theta =\frac{2\pi }{L}$. Finally, n∈C denotes the additive white Gaussian noise at the user with zero mean and variance $\sigma^{2}$.

### 2.2. Channel Model

The BS-IRS channel and IRS-user channel are both formulated as Rician fading channels, each consisting of a line-of-sight (LoS) and a non-line-of-sight (NLoS) component. The (m,n)-th element of the BS-IRS channel matrix G can be expressed as

(2) $${\left[G\right]}_{m,n}=\sqrt{\alpha d_{m,n}^{-\beta }}\left(\sqrt{\frac{\kappa }{\kappa +1}}{\left[G_{\mathrm{LoS}}\right]}_{m,n}+\sqrt{\frac{1}{\kappa +1}}{\left[G_{\mathrm{NLoS}}\right]}_{m,n}\right),$$

where κ denotes the Rician factor, α denotes the large-scale path loss at the reference distance 1 m, β denotes the path loss exponent, and $d_{m,n}$ denotes the distance between the *m*-th element of the IRS and the *n*-th antenna of the BS. The (m,n)-th element of $G_{\mathrm{LoS}}$ can be represented as

(3) $${\left[G_{\mathrm{LoS}}\right]}_{m,n}=\exp\left(\frac{-j2\pi d_{m,n}}{\lambda }\right).$$

For the component of $G_{\mathrm{NLoS}}$, each element is assumed to be independently and identically distributed as ${\left[G_{\mathrm{NLoS}}\right]}_{m,n}∼\mathrm{CN}\left(0,1\right)$.

The IRS-user channel h is modeled similarly with the associated parameters, and the (m)-th element of h is expressed as

(4) $${\left[h\right]}_{m}=\sqrt{\alpha {\hat{d}}_{m}^{-\beta }}\left(\sqrt{\frac{\kappa }{\kappa +1}}{\left[h_{\mathrm{LoS}}\right]}_{m}+\sqrt{\frac{1}{\kappa +1}}{\left[h_{\mathrm{NLoS}}\right]}_{m}\right),$$

where ${\hat{d}}_{m}$ denotes the distance between the *m*-th element of the IRS and the antenna of the user.

### 2.3. Power Model

The total power consumption of the system is given by

(5) $$P_{\mathrm{all}}=P_{\mathrm{BS}}+P_{\mathrm{IRS}}+P_{\mathrm{static}},$$

where $P_{\mathrm{BS}}=f^{H}f$ is the power consumption of the transmitted signals at the BS, related to the beamforming designs of the BS. $P_{\mathrm{IRS}}$ is the PS-DPC at the PIN diode-based IRS, related to the phase shift matrix of the IRS and derived from a practical model. $P_{\mathrm{static}}$ contains other static power consumption terms in the system, e.g., the BS contains the power consumption of the baseband processing, cooling, circuit synchronization, radio frequency chains, etc. The IRS contains the power consumption of the field-programmable gate array (FPGA) and control circuits. However, as these power consumption terms are constant regardless of the beamforming designs, we omit these terms and only consider the dynamic power consumption in this paper, as similar to that in [39].

In the analysis of the PS-DPC model of the IRS, we consider a *b*-bit IRS, where each IRS element consists of *b* PIN diodes and can realize $L=2^{b}$ possible phase shifts by controlling the on/off-state of the corresponding PIN diodes. The phase shift of the IRS element satisfies $\mathrm{Arg}\left\{{\left[\Phi \right]}_{m,m}\right\}=\theta_{m}\in \left\{0,\Delta \theta ,\ldots ,\left(L-1\right)\Delta \theta \right\}$, where $\Delta \theta =\frac{2\pi }{L}$. For each PIN diode, the on-state is encoded as “1” and the power consumption is $P_{\mathrm{PIN}}$, whereas the off-state is encoded as “0” and is power-free [38]. The state of the *m*-th element can be encoded as a binary codeword $\beta_{m}$ with *b* bits. Therefore, the reflection coefficient of the *m*-th element corresponding to $\beta_{m}$ can be written as

(6) $${\left[\Phi \right]}_{m,m}=F\left(\beta_{m}\right).$$

The power consumption of the *m*-th element is represented as

(7) $$P_{m}=G\left(\beta_{m}\right)=P_{\mathrm{PIN}}1_{b}^{T}\beta_{m}.$$

To clarify the explanation, we present an example of a 2-bit IRS, in which the reflection coefficient and power consumption of the *m*-th element are defined as follows:

(8) $$\begin{array}{ccc} {\left[\Phi \right]}_{m,m}=e^{j0}, & P_{m}=0, & \mathrm{if}\;\beta_{m}={\left[0,0\right]}^{T},\\ {\left[\Phi \right]}_{m,m}=e^{j\frac{\pi }{2}}, & P_{m}=P_{\mathrm{PIN}}, & \mathrm{if}\;\beta_{m}={\left[0,1\right]}^{T},\\ {\left[\Phi \right]}_{m,m}=e^{j\pi }, & P_{m}=P_{\mathrm{PIN}}, & \mathrm{if}\;\beta_{m}={\left[1,0\right]}^{T},\\ {\left[\Phi \right]}_{m,m}=e^{j\frac{3\pi }{2}}, & P_{m}=2P_{\mathrm{PIN}}, & \mathrm{if}\;\beta_{m}={\left[1,1\right]}^{T}, \end{array}$$

It can be noted that the relationship between ${\left[\Phi \right]}_{m,m}$ and $\beta_{m}$ is implicit. Hence, we will further address these issues in Section 3 and Section 4.

### 2.4. Problem Formulation

To minimize the total power consumption with the practical PS-DPC model, the beamforming optimization problem can be formulated as

(9) $$\begin{array}{cc} \min_{f,\Phi } & P_{\mathrm{all}}=P_{\mathrm{BS}}+P_{\mathrm{IRS}}=f^{H}f+P\left(\Phi \right)\\ s.t. & C1\;:\;R⩾R_{\mathrm{set}},\\ & C2:\;{\left[\Phi \right]}_{m,m}\in \left\{e^{j0},e^{j\Delta \theta },\ldots ,e^{j(L-1)\Delta \theta }\right\},\;\forall m, \end{array}$$

where $P_{\mathrm{IRS}}=P\left(\Phi \right)$, $R=\log\left[1+\left(h^{H}\Phi Gff^{H}G^{H}\Phi^{H}h\right)/\sigma^{2}\right]$ is the achievable rate, $R_{\mathrm{set}}$ is the minimum required rate of the user, and constraint C1 represents the QoS requirement. Even though we already know the relationship between ${\left[\Phi \right]}_{m,m}$ and $\beta_{m}$, $P_{m}$ and $\beta_{m}$, it is difficult to explicitly represent $P_{\mathrm{IRS}}=P\left(\Phi \right)$ because the relationship between $P_{m}$ and ${\left[\Phi \right]}_{m,m}$ is nonlinear when b⩾2, which will be further discussed in Section 3 and Section 4.

## 3. Beamforming Optimization for the Single-User Scenario

As can be observed in problem (9), it is intractable because of the implicit and nonlinear relationship in $P_{\mathrm{IRS}}=P\left(\Phi \right)$. In this section, we first introduce a selection matrix to explicitly represent the PS-DPC and the phase shift matrix. Then, we propose a GBD-based algorithm to achieve beamforming optimization at the BS and IRS jointly.

### 3.1. Solving the Nonlinear Relationship in $P_{\mathrm{IRS}}=P\left(\Phi \right)$

To deal with the implicit and nonlinear relationship in $P_{\mathrm{IRS}}=P\left(\Phi \right)$, notice that the essence of optimizing the phase shift for a reflection element is to select the possible states of its PIN diodes. Hence, we introduce a selection vector $c_{m}$ to directly represent the state of the *m*-th element, thereby we can explicitly represent $P_{\mathrm{IRS}}$ and Φ. Specifically, $c_{m}$ satisfies these following conditions:

(10) $${\left[c_{m}\right]}_{l}\in \left\{0,1\right\},1_{L}^{T}c_{m}=1,l\in L=\left\{1,2,\ldots ,L\right\}.$$

Taking the 2-bit IRS as an example, $c_{m}$ are defined as

(11) $$c_{m}=\left\{\begin{array}{cc} {\left[1,0,0,0\right]}^{T}, & \beta_{m}={\left[0,0\right]}^{T}\\ {\left[0,1,0,0\right]}^{T}, & \beta_{m}={\left[0,1\right]}^{T}\\ {\left[0,0,1,0\right]}^{T}, & \beta_{m}={\left[1,0\right]}^{T}\\ {\left[0,0,0,1\right]}^{T}, & \beta_{m}={\left[1,1\right]}^{T} \end{array}\right..$$

We further introduce a reflection coefficient vector $a={\left[e^{j0},e^{j\Delta \theta },\ldots ,e^{j(L-1)\Delta \theta }\right]}^{T}\in C^{L\times 1}$ that includes *L* possible reflection coefficients and a power consumption vector $p\in C^{L\times 1}$ that includes the power consumption of *L* corresponding phase shifts. For example, we have $a={\left[e^{j0},e^{j\frac{\pi }{2}},e^{j\pi },e^{j\frac{3\pi }{2}}\right]}^{T}$ and $p={\left[0,P_{\mathrm{PIN}},P_{\mathrm{PIN}},2P_{\mathrm{PIN}}\right]}^{T}$ for the 2-bit IRS.

Hence, the reflection coefficient and the power consumption of the *m*-th element can be expressed as ${\left[\Phi \right]}_{m,m}=a^{T}c_{m}$ and $P_{m}=p^{T}c_{m}$, respectively. Thus, Φ and $P_{\mathrm{IRS}}$ can be further expressed as

(12) $$\Phi =\mathrm{AC},\;P_{\mathrm{IRS}}=\mathrm{Tr}\left(\mathrm{PC}\right),$$

where $C\in C^{LM\times M}$, $A\in C^{M\times LM}$, and $P\in C^{M\times LM}$ are the block diagonal matrix, defined as follows:

(13) $$C=\left[\begin{array}{cccc} c_{1} & 0 & \ldots & 0\\ 0 & c_{2} & \ldots & 0\\ \vdots & \vdots & \ddots & \vdots \\ 0 & 0 & \ldots & c_{M} \end{array}\right],\;A=\left[\begin{array}{cccc} a^{T} & 0^{T} & \ldots & 0^{T}\\ 0^{T} & a^{T} & \ldots & 0^{T}\\ \vdots & \vdots & \ddots & \vdots \\ 0^{T} & 0^{T} & \ldots & a^{T} \end{array}\right],\;P=\left[\begin{array}{cccc} p^{T} & 0^{T} & \ldots & 0^{T}\\ 0^{T} & p^{T} & \ldots & 0^{T}\\ \vdots & \vdots & \ddots & \vdots \\ 0^{T} & 0^{T} & \ldots & p^{T} \end{array}\right].$$

By introducing the selection matrix C, problem (9) is equivalently reformulated as

(14) $$\begin{array}{cc} \min_{f,C} & P_{\mathrm{all}}=P_{\mathrm{BS}}+P_{\mathrm{IRS}}=f^{H}f+\mathrm{Tr}\left(\mathrm{PC}\right)\\ s.t. & C1\;:\;R⩾R_{\mathrm{set}},\\ & C2a:\;{\left[c_{m}\right]}_{l}\in \left\{0,1\right\},\;\forall m,\;\forall l,\\ & C2b:\;1_{L}^{T}c_{m}=1,\;\forall m, \end{array}$$

where $R=\log\left[1+\left(h^{H}\mathrm{AC}Gff^{H}G^{H}C^{H}A^{H}h\right)/\sigma^{2}\right]$, and constraints C2a and C2b are equivalent to constraint C2 in problem (9). Based on the known A and P, the selection matrix C contains all the phase shift information and power consumption information of the IRS. C has the linear relationship with both Φ and $P_{\mathrm{IRS}}$. Thus, the difficulty of the implicit relationship in $P_{\mathrm{IRS}}=P\left(\Phi \right)$ is solved by optimizing C instead of Φ.

### 3.2. Generalized Benders Decomposition Algorithm

Due to the coupling of f and C in constraint C1, and the binary constraint of $c_{m}$, problem (14) is complicated and challenging. Thus, we further transform this MINLP problem and propose a GBD-based algorithm.

To apply the GBD algorithm, the optimization problem should satisfy the following framework [42]:

- If the discrete variables are fixed, the optimization problem should be convex to the continuous variables.
- If the continuous variables are fixed, the optimization problem should be linear to the discrete variables.

Unfortunately, as constraint C1 is not convex with respect to f, we cannot directly apply the GBD algorithm to solve problem (14). To deal with this issue, we first rewrite constraint C1 as

(15) $$h^{H}\mathrm{AC}Gff^{H}G^{H}C^{H}A^{H}h⩾\gamma ,$$

where $\gamma =\left(2^{R_{\mathrm{set}}}-1\right)\sigma^{2}$. Furthermore, for any optimal solution $\hat{f}$ and any phase shift α, $e^{j\alpha }\hat{f}$ is an optimal solution as well. Hence, problem (14) can be expressed as

(16) $$\begin{array}{cc} \min_{f,C} & P_{\mathrm{all}}=f^{H}f+\mathrm{Tr}\left(\mathrm{PC}\right)\\ s.t. & C1a:\;h^{H}\mathrm{AC}Gf⩾\sqrt{\gamma },\\ & C1b:\;h^{H}\mathrm{AC}Gf-{\left(h^{H}\mathrm{AC}Gf\right)}^{*}=0,\\ & C2a:\;c_{m}\left[l\right]\in \left\{0,1\right\},\;\forall m,\;\forall l,\\ & C2b:\;1_{L}^{T}c_{m}=1,\;\forall m, \end{array}$$

where constraint C1 is equivalently expressed by constraints C1a and C1b. Now, problem (16) satisfies the framework of the GBD algorithm.

#### 3.2.1. Primal Problem

In the (i)-th iteration, the primal problem is to optimize f, expressed as

(17) $$\begin{array}{cc} \min_{f} & f^{H}f+\mathrm{Tr}\left(PC^{\left(i-1\right)}\right)\\ s.t. & C1a:\;h^{H}AC^{\left(i-1\right)}Gf⩾\sqrt{\gamma },\\ & C1b:\;h^{H}AC^{\left(i-1\right)}Gf-{\left(h^{H}AC^{\left(i-1\right)}Gf\right)}^{*}=0, \end{array}$$

where $C^{\left(i-1\right)}$ is the selection matrix from the (i−1)-th iteration. Problem (17) is a convex problem that can be solved by the convex problem solvers, such as CVX [43]. By solving the primal problem, we obtain the optimal solution $f^{\left(i\right)}$. Based on the known $C^{\left(i-1\right)}$ and the obtained solution $f^{\left(i\right)}$, the primal problem can provide an upper bound (UB) of problem (16), expressed as

(18) $$\eta_{UB}^{\left(i\right)}=P_{\mathrm{all}}^{\left(i\right)}={f^{\left(i\right)}}^{H}f^{\left(i\right)}+\mathrm{Tr}\left(PC^{\left(i-1\right)}\right).$$

In addition, to formulate the master problem, the Lagrangian function of the primal problem is expressed as

(19) $$\begin{array}{cc} \; & L\left(f,C^{\left(i-1\right)},\lambda ,\nu \right)=f^{H}f+\mathrm{Tr}\left(PC^{\left(i-1\right)}\right)+\lambda \left(-h^{H}AC^{\left(i-1\right)}Gf+\sqrt{\gamma }\right)\\ \; & \;\;\;\;\;\;+\nu [h^{H}AC^{\left(i-1\right)}Gf-{\left(h^{H}AC^{\left(i-1\right)}Gf\right)}^{*}], \end{array}$$

where λ and ν are the dual variables of constraints C1a and C1b, respectively, and the optimal dual variables in the (i)-th iteration are $\lambda^{\left(i\right)}$ and $\nu^{\left(i\right)}$.

#### 3.2.2. Master Problem

The formulation of the master problem is based on the nonlinear convex duality theory [42]. Specifically, the master problem in the (i)-th iteration can be expressed as

(20) $$\begin{array}{cc} \min_{C,\eta_{LB}} & \eta_{LB}\\ s.t. & C2a:\;c_{m}\left[l\right]\in \left\{0,1\right\},\;\forall m,\;\forall l,\\ & C2b:\;1_{L}^{T}c_{m}=1,\;\forall m,\\ & C3\;:\;\eta_{LB}⩾\min_{f}L(f,C,\lambda^{\left(t\right)},\nu^{\left(t\right)}),\;\forall t\in T, \end{array}$$

where $\eta_{LB}$ is an auxiliary optimization variable representing the lower bound (LB), and set $T=\left\{1,2,\ldots ,i\right\}$ contains all of the sequence numbers in the previous *i* iterations. Note that problem (20) is still hard to solve because of constraint C3, in which we should optimize the variable f as well. To tackle this issue, we further transform (20) to the variant 2 (V-2) form of the GBD algorithm [42] as follows:

(21) $$\begin{array}{cc} \min_{C,{\tilde{\eta }}_{LB}} & {\tilde{\eta }}_{LB}\\ s.t. & C2a:\;c_{m}\left[l\right]\in \left\{0,1\right\},\;\forall m,\;\forall l,\\ & C2b:\;1_{L}^{T}c_{m}=1,\;\forall m,\\ & \tilde{C3}\;:\;{\tilde{\eta }}_{LB}⩾L\left(f^{\left(t\right)},C,\lambda^{\left(t\right)},\nu^{\left(t\right)}\right),\;\forall t\in T, \end{array}$$

in which we replace the optimization problem about f in constraint C3 with the Lagrangian function in constraint $\tilde{C3}$ and fix f to $f^{\left(t\right)}$ obtained in the (t)-th iteration.

Now, the master problem becomes a mixed-integer linear programming (MILP) problem and can be solved by solvers for MILPs, such as MOSEK. By solving the master problem, we obtain the selection matrix $C^{\left(i\right)}$ for the next iteration and the optimal value ${\tilde{\eta }}_{LB}$ as the LB of the original problem (16). Finally, by iteratively solving the primal and master problems, the gap between the UB and LB will be reduced, and the solution will converge. The proposed GBD algorithm is summarized in Algorithm 1.
**Algorithm 1** The GBD-based algorithm for the multi-bit IRS with the PS-DPC model in the single-user scenario.1:Set i=0, $\eta_{UB}^{\left(0\right)}=\infty$, ${\tilde{\eta }}_{LB}^{\left(0\right)}=-\infty$ and tolerance Δ;2:Randomly initialize $C^{\left(0\right)}$;3:**repeat**4:   i←i+1;5:   Solve the primal problem (17) with $C^{\left(i-1\right)}$;6:   Obtain $f^{\left(i\right)}$, $\lambda^{\left(i\right)}$, $\nu^{\left(i\right)}$ and $\eta_{UB}^{\left(i\right)}$;7:   Construct $L(f^{\left(i\right)},C,\lambda^{\left(i\right)},\nu^{\left(i\right)})$ according to (19);8:   Solve the master problem (21);9:   Obtain $C^{\left(i\right)}$ and ${{\tilde{\eta }}_{LB}}^{\left(i\right)}$;10:**until** $\eta_{UB}^{\left(i\right)}-{\tilde{\eta }}_{LB}^{\left(i\right)}⩽\Delta$

## 4. Beamforming Optimization for the Multi-User Scenario

### 4.1. Problem Formulation

In the multi-user scenario, we further consider *K* single-antenna users. The other devices are the same as in the single-user scenario. The received signal at the *k*-th user can be expressed as

(22) $$y_{k}=h_{k}^{H}\Phi Gf_{k}s_{k}+\sum_{i\neq k}^{K}h_{k}^{H}\Phi Gf_{i}s_{i}+n_{k},$$

where $s={\left[s_{1},s_{2},\ldots ,s_{K}\right]}^{T}\in C^{K\times 1}$ denotes the original symbol vector with $E\left\{ss^{H}\right\}=I_{K}$, and $F=\left[f_{1},f_{2},\ldots ,f_{K}\right]\in C^{N\times K}$ denotes the digital beamforming matrix at the BS. The BS-IRS channel and IRS-${\mathrm{user}}_{k}$ channel are modeled as Rician fading channels, expressed as $G\in C^{M\times N}$ and $h_{k}\in C^{M\times 1}$, respectively. $n_{k}\in C$ denotes the additive white Gaussian noise at the *k*-th user with zero mean and variance ${\sigma_{k}}^{2}$.

To minimize the total power consumption of the system, the optimization problem of the multi-user scenario is formulated as

(23) $$\begin{array}{cc} \min_{F,\Phi } & P_{\mathrm{all}}=P_{\mathrm{BS}}+P_{\mathrm{IRS}}=\mathrm{Tr}\left(FF^{H}\right)+P\left(\Phi \right)\\ s.t. & C4\;:\;R_{k}⩾R_{\mathrm{set},k},\;\forall k,\\ & {C2\;:\;[\Phi ]}_{m,m}\in \left\{e^{j0},e^{j\Delta \theta },\ldots ,e^{j(L-1)\Delta \theta }\right\},\;\forall m, \end{array}$$

where constraint C4 represents the QoS requirement, $R_{\mathrm{set},k}$ is the minimum required rate of the *k*-th user, and $R_{k}$ is the achievable rate at the *k*-th user given by

(24) $$R_{k}=\log\left(1+\frac{h_{k}^{H}\Phi Gf_{k}f_{k}^{H}G^{H}\Phi^{H}h_{k}}{\sum_{i\neq k}^{K}h_{k}^{H}\Phi Gf_{i}f_{i}^{H}G^{H}\Phi^{H}h_{k}+\sigma_{k}^{2}}\right).$$

Similar to that in Section 3.1, problem (23) is intractable because P(Φ) can not be expressed explicitly. Therefore, we introduce the selection matrix C to explicitly represent the PS-DPC and the phase shift matrix as well. According to the matrix C, A, and P defined in (13), problem (23) can also be equivalently reformulated as

(25) $$\begin{array}{cc} \min_{F,C} & P_{\mathrm{all}}=P_{\mathrm{BS}}+P_{\mathrm{IRS}}=\mathrm{Tr}\left(FF^{H}\right)+\mathrm{Tr}\left(\mathrm{PC}\right)\\ s.t. & C4\;:\;R_{k}⩾R_{\mathrm{set},k},\;\forall k,\\ & C2a\;:\;{\left[c_{m}\right]}_{l}\in \left\{0,1\right\},\;\forall m,\;\forall l,\\ & C2b\;:\;1_{L}^{T}c_{m}=1,\;\forall m, \end{array}$$

where constraints C2a and C2b are equivalent to constraint C2 in problem (23), and the achievable rate $R_{k}$ is expressed as

(26) $$R_{k}=\log\left(1+\frac{h_{k}^{H}\mathrm{AC}Gf_{k}f_{k}^{H}G^{H}C^{H}A^{H}h_{k}}{\sum_{i\neq k}^{K}h_{k}^{H}\mathrm{AC}Gf_{i}f_{i}^{H}G^{H}C^{H}A^{H}h_{k}+\sigma_{k}^{2}}\right).$$

### 4.2. Coordinate Descent Algorithm

Although the transformation solves the nonlinear and implicit relationship in P(Φ), problem (25) remains challenging due to the coupled variables in the fractional constraint C4 and the binary variables in constraint C2a. Thus, we propose a CD-based heuristic algorithm to optimize the selection matrix of the IRS iteratively.

In the CD algorithm, for the *m*-th element of a *b*-bit IRS, we set ${\left[c_{m}\right]}_{l}=1$ for each l∈L, respectively, while fixing the other selection vectors $c_{n}\;\left(n\neq m\right)$. In the process, we design the corresponding beamforming matrix and calculate the corresponding total power consumption for $L=2^{b}$ different selection vectors $c_{m}$. Finally, the current optimal selection vector of the *m*-th element ${\hat{c}}_{m}$ is chosen by comparing the corresponding total power consumption, which can be expressed as

(27) $$\begin{array}{cc} \hat{F},{\hat{c}}_{m}=\arg\;\min_{F,c_{m}} & \mathrm{Tr}\left(FF^{H}\right)+\mathrm{Tr}\left(\mathrm{PC}\right)\\ s.t.\;\;\;\;\; & C4\;:\;R_{k}⩾R_{\mathrm{set},k},\forall k,\\ & C2a\;:\;{\left[c_{m}\right]}_{l}\in \left\{0,1\right\},\;\forall l,\\ & C2b\;:\;1_{L}^{T}c_{m}=1, \end{array}$$

where F is optimized with a fixed C. The problem of F can be expressed as

(28) $$\begin{array}{cc} \min_{F} & \mathrm{Tr}(FF^{H})\\ s.t. & \frac{h_{k}^{H}\mathrm{AC}Gf_{k}f_{k}^{H}G^{H}C^{H}A^{H}h_{k}}{\sum_{i\neq k}^{K}h_{k}^{H}\mathrm{AC}Gf_{i}f_{i}^{H}G^{H}C^{H}A^{H}h_{k}+\sigma_{k}^{2}}⩾\omega_{k},\;\forall k, \end{array}$$

where $\omega_{k}=2^{R_{\mathrm{set},k}}-1$, and F can be designed via the zero-forcing method, expressed as

(29) $$F=H_{e}^{H}{\left(H_{e}H_{e}^{H}\right)}^{-1}P^{\frac{1}{2}},$$

where $H_{e}=H\mathrm{AC}G$ is the equal channel matrix with $H={\left[h_{1},\ldots ,h_{K}\right]}^{H}$. The power allocation matrix is $P=\mathrm{diag}(p_{1},\ldots ,p_{K})$ with $p_{k}={\sigma_{k}}^{2}\omega_{k}$.

The beamforming optimization problem under the PS-DPC has been solved by the CD algorithm, summarized in Algorithm 2. In the CD algorithm, the power consumption is monotonically decreasing by iteratively optimizing the phase shift of each element. Hence, the CD algorithm will converge to a global or local optimal solution after several iterations. Assuming that the number of iterations is $I_{CD}$, we usually set $I_{CD}=9$ in the simulation experiments. The complexity of updating the beamforming matrix F is $O\left(KLM^{2}\right)$, and the complexity of the CD algorithm is $O\left(I_{CD}KL^{2}M^{3}\right)$.
**Algorithm 2** The CD-based algorithm for the multi-bit IRS with the PS-DPC model in the multi-user scenario.1:Set i=0, $I_{CD}$;2:Randomly initialize C;3:**repeat**4:   i←i+1;5:   m=1;6:   **repeat**7:     Loop through each phase shift for (m)-th element and get the corresponding beamforming matrix F according to (29);8:     Compare and get $\hat{F}$ and ${\hat{c}}_{m}$ with the lowest power consumption according to problem (27);9:     m←m+1.10:   **until** m=M11:**until** $i=I_{CD}$ or the stopping condition is satisfied.

### 4.3. Genetic Algorithm

Although the CD algorithm proposed in Section 4.2 is a feasible and efficient scheme to solve the MINLP problem, there are numerous operations to optimize each IRS element in several iterations. Therefore, we further investigate and propose a genetic algorithm to reduce the computation complexity.

The genetic algorithm is an optimization scheme inspired by genetics. Similar to the natural biological evolution and genetic selections, the genetic algorithm begins with a set of feasible solutions and then implements the genetic operations, e.g., mutation and crossover. After these operations, the best individuals are selected from the new generation and cross into the next generation. Over several generations, the individuals will converge toward the global or local optimal solutions.

Genetic algorithms have particular advantages for solving complex optimization problems, while some traditional algorithms may perform inefficiently. The characteristic of evolutionary principles gives the genetic algorithm the ability to efficiently explore large search spaces and adapt to dynamic environments. In addition, the genetic algorithm has fewer requirements regarding problem formulation and constraint conditions. Therefore, genetic algorithms can be widely used in a wide range of applications, such as wireless communications [44,45].

According to problem (25), we set the selection matrix C as the population in the genetic algorithm. For each individual C, we optimize the beamforming matrix F and calculate the fitness function. By comparing the fitness of each individual C, the population is renewed and crossed into the next generation. Over several iterations until the stopping condition is satisfied, the final optimization C and corresponding F are obtained. The steps of the proposed genetic algorithm are shown in detail as follows:

1. Initial population generation: We randomly generate ρ individual C, satisfying constraints C2a and C2b. For the selection vector $c_{m},\forall m$ is the gene of C. For each selection matrix C, the corresponding beamforming matrix F is designed via the zero-forcing method according to (29).
2. Fitness evaluation: For the existing population, we evaluate the fitness value f(C,F) of each individual, expressed as
  (30) $$f\left(C,F\right)=-P_{\mathrm{all}}=-\mathrm{Tr}\left(FF^{H}\right)-\mathrm{Tr}\left(\mathrm{PC}\right),$$
  which is the opposite value of the optimization objective function in problem (25).
3. Selection: A larger fitness value indicates that the corresponding system power consumption is lower. According to the fitness value of each individual C, we select $\frac{\rho }{4}$ individuals that have the maximum fitness value as the parents.
4. Crossover and mutation: We implement the genetic operators for the $\frac{\rho }{4}$ parents, i.e., crossover and mutation. In the crossover operator, we choose two parents and exchange some of their genes. Specifically, the crossover operator includes the single-point crossover and uniform crossover. We assume that two parents $C_{a}$ and $C_{b}$ are expressed as
  (31) $$\begin{array}{cc} \; & C_{a}=\mathrm{diag}\left(c_{a,1},\ldots ,c_{a,m},\ldots ,c_{a,M}\right),\\ \; & C_{b}=\mathrm{diag}\left(c_{b,1},\ldots ,c_{b,m},\ldots ,c_{b,M}\right). \end{array}$$
  For the single-point crossover, we set a random value *m* and exchange the genes of $C_{a}$ and $C_{b}$ at the *m*-th element, that is, the new individuals ${\tilde{C}}_{a}$ and ${\tilde{C}}_{b}$ are expressed as
  (32) $$\begin{array}{cc} \; & {\tilde{C}}_{a}=\mathrm{diag}\left(c_{a,1},\ldots ,c_{a,m},c_{b,m+1},\ldots ,c_{b,M}\right),\\ \; & {\tilde{C}}_{b}=\mathrm{diag}\left(c_{b,1},\ldots ,c_{b,m},c_{a,m+1},\ldots ,c_{a,M}\right). \end{array}$$
  For the uniform crossover, the two parents randomly exchange the *m*-th selection vector for all *m* and become the new individuals. In the mutation operator, we choose a parent and randomly change some of their genes as the new individual.
5. New population generation: The new population consists of $\frac{\rho }{4}$ parents, $\frac{\rho }{4}$ new individuals generated by the single-point crossover, $\frac{\rho }{4}$ new individuals generated by the uniform crossover, and $\frac{\rho }{4}$ new individuals generated by the mutation operator. Hence, there are still ρ individuals C in the population. Then, we further evaluate the fitness value of this population in step 2 and repeat the above operators until the stopping condition is satisfied.

The power minimization problem under the PS-DPC has been solved by the genetic algorithm, summarized in Algorithm 3. By iteratively generating and selecting the individuals, the power consumption of the system decreases in each iteration. The genetic algorithm will converge to a global or local optimal solution after several iterations. Assuming that the number of iterations is $I_{GA}$, the complexity of the genetic algorithm is $O\left(\frac{3}{4}I_{GA}\rho KLM^{2}\right)$. In the simulation experiments, we usually set $I_{GA}=3\rho$ and $\rho =\sqrt{2LM}$, and the specific complexity is $O\left(\frac{9}{2}KL^{2}M^{3}\right)$, which is half of the CD algorithm.
**Algorithm 3** The genetic algorithm for the multi-bit IRS with the PS-DPC model in the multi-user scenario.1:Set i=0, $I_{CD}$;2:Randomly generate ρ initial C;3:**repeat**4:   i←i+1;5:   Calculate and evaluate the fitness value of the individuals in the existing population according to (30);6:   Select $\frac{\rho }{4}$ individuals that has the maximum fitness value as the parents;7:   Implement the genetic operators for the parents, including the single-point crossover, the uniform crossover and the mutation operator.8:   Get the new population generation.9:**until** $i=I_{GA}$ or the stopping condition is satisfied.

## 5. Simulation Results

### 5.1. Simulation Setup

We consider a downlink system, where the BS is equipped with a uniform linear array of N=3 antennas in the single-user scenario and N=2K antennas in the multi-user scenario. The IRS is a uniform square planar array and is 40 m away from the BS. The users are uniformly distributed in the region where the distance between the IRS and users is from 2 m to 20 m. The channel models are introduced in Section 2, where the Rician factor κ=3, the large-scale path loss $\alpha =10^{-4}$, and the path loss exponent β=2.2. The carrier frequency is set to 3.5GHz. The element separation of the BS and IRS are both set to 0.5λ. The power consumption of a PIN diode in the on-state is set to $P_{\mathrm{PIN}}=12\;\mathrm{mW}$ [38]. The noise variance of each user is −110dBm.

For comparison, we also provide the simulation results of two baseline algorithms, i.e., the RAND and NPS algorithms. In the former, the IRS phase shift matrix is initialized randomly without optimization. In the latter one, for the single-user scenario, a similar GBD algorithm is employed without considering the power consumption constraint in the problem. For the multi-user scenario, a similar CD algorithm is employed without considering the power consumption constraint in the problem. However, the actually consumed power of the IRS is counted in the total system power consumption in the simulation. It should be noted that the NPS algorithm is based on our proposed algorithm with some minor modifications. Hence, the NPS algorithm can also be regarded as our proposed algorithm. In addition, the simulation results of the NPS algorithm highlight the beamforming capability of our approach. The simulation results of the GBD and CD algorithms reflect more on the capability of our approach to further reduce power consumption.

### 5.2. Single-User Scenario

**Figure 2:**
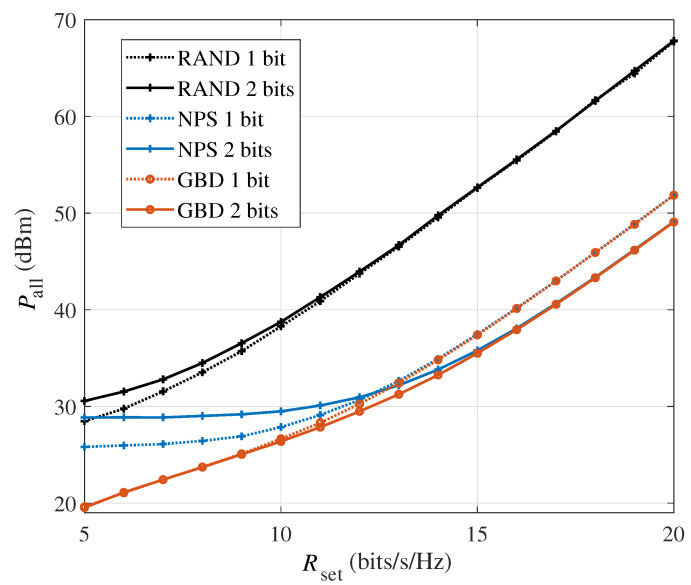
The system power consumption versus the rate requirement $R_{\mathrm{set}}$ in the single-user scenario (M=64).

Figure 2 shows the total system power consumption versus the minimum required rate $R_{\mathrm{set}}$ in the single-user scenario with M=64. As can be observed, the power consumption of all the algorithms increases with the growth of $R_{\mathrm{set}}$. Since the RAND algorithm randomly chooses the phase shifts of all the IRS elements without optimization, it has the highest power consumption. The NPS algorithm, on the other hand, tends to improve the IRS beamforming quality as much as possible. Therefore, it always performs better than the RAND algorithm. However, as it ignores the PS-DPC during the optimization, the NPS algorithm leads to the power consumption on a high level. Compared with the baseline algorithms, the proposed GBD algorithm can effectively reduce the power consumption to the best effect. However, the proposed GBD algorithm has a gain over the NPS algorithm at a low $R_{\mathrm{set}}$, while their performance is almost identical at a higher $R_{\mathrm{set}}$. The reason for this phenomenon is the upper limit of the IRS power consumption, which will be further explained later. Meanwhile, the power consumption of the GBD algorithm is better in the 2-bit IRS scenario than in the 1-bit one, which is more obvious at a higher $R_{\mathrm{set}}$. Hence, on the one hand, it is necessary to consider the PS-DPC of the IRS, especially in cases with low rate requirements; otherwise, improving the phase shift resolution of the IRS may be invalid or cause more power consumption. On the other hand, if the system requires a high rate, improving the phase shift quantization bits can help the system save power consumption.

**Figure 3:**
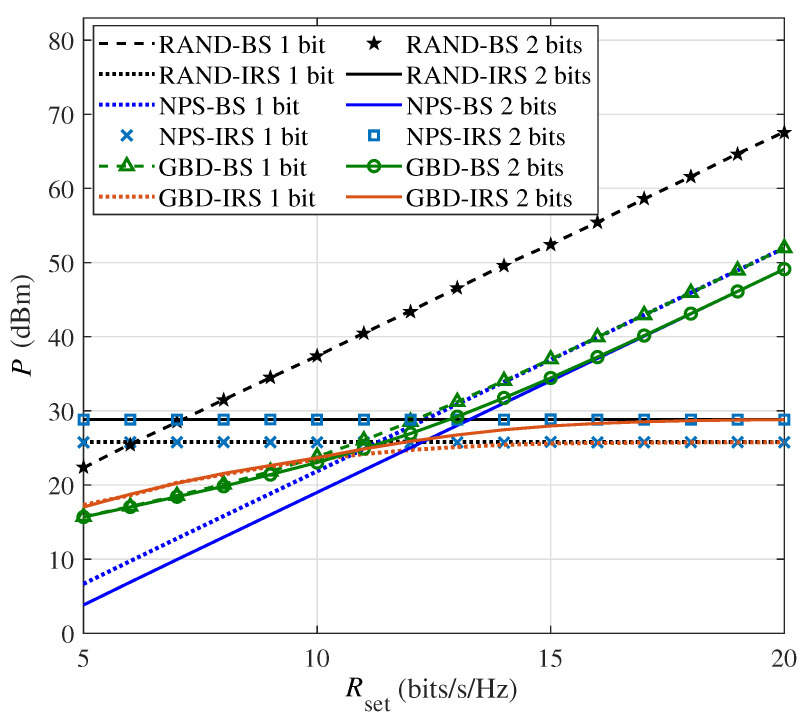
The BS and IRS power consumption versus the rate requirement $R_{\mathrm{set}}$ in the single-user scenario (M=64).

Figure 3 shows the BS and IRS power consumption versus the minimum required rate $R_{\mathrm{set}}$ in the single-user scenario with M=64 in order to further analyze the phenomenon in Figure 2. Firstly, for the RAND algorithm, the BS power consumption is the highest of the three algorithms. As the IRS phase shifts are designed randomly, the IRS can not effectively concentrate the transmit signal at the user, and the required rate can only be ensured by the high power consumption of the BS. For each IRS element, the probability of each possible phase shift is the same. In simulations that generate multiple channels, the average IRS element power consumption is the average of the power consumption corresponding to all possible phase shifts. For different required rates, the power consumption of the IRS is constant and is equal to the average power consumption of all possible phase shifts for one element multiplied by the number of IRS elements. In addition, the 2-bit IRS power consumption is higher than the 1-bit IRS, because the average power consumption of 2-bit elements is higher. Secondly, for the NPS algorithm, the performance of the BS is lower than the RAND algorithm; this is because the IRS in the NPS algorithm has the ability to optimize the beamforming, thereby reducing the load on the BS. Due to the ignoring of the PS-DPC in the NPS algorithm, the designing of the phase shift matrix only aims to maximize the beamforming capabilities of the IRS. Therefore, in multiple different channels, each phase shift has the same probability of occurrence. Hence, the performance of the IRS power consumption is similar to the RAND algorithm. However, because the NPS and RAND algorithms ignore the PS-DPC, the IRS power consumption is always at a high value, resulting in high total power consumption. Thirdly, for the GBD algorithm, the BS and IRS power consumption is related to the required rate. As the GBD algorithm considers the PS-DPC of the IRS and aims to minimize the total system power consumption, it can balance the BS and IRS power consumption. Specifically, the GBD algorithm tends to make the BS and IRS each bear half of the power consumption at a low required rate. While the required rate is high, the GBD algorithm tends to make the IRS provide a high beamforming gain. At this point, the BS supplies significant transmission power to meet the rate requirement, making its power consumption the dominant factor in the system. It can be seen by comparing Figure 2; if the power consumption of the BS is less than or comparable to the IRS, the GBD algorithm has a gain over the NPS algorithm. In addition, if the user requires a low rate, the 1-bit and 2-bit IRSs have similar power consumption, which is consistent with the performance and conclusion in Figure 2.

**Figure 4:**
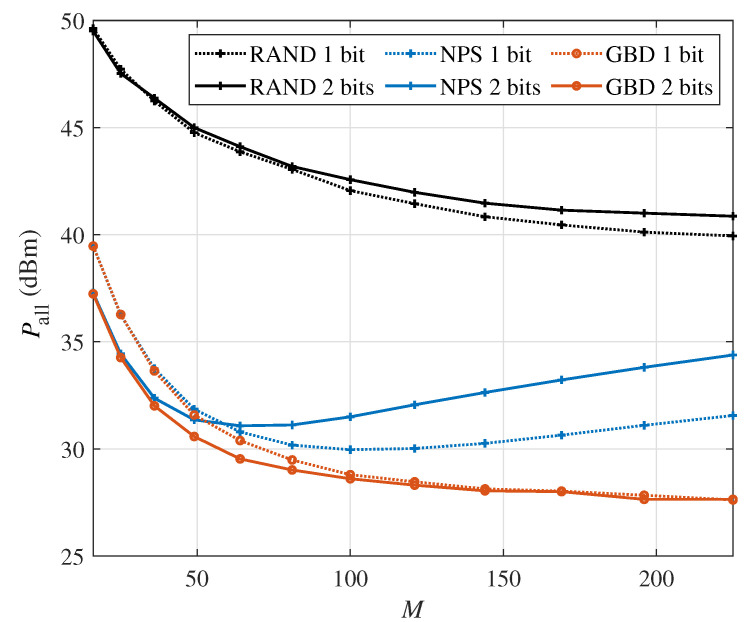
The system power consumption versus the number of IRS reflection elements *M* in the single-user scenario ($R_{\mathrm{set}}=12\;\mathrm{bits}/s/\mathrm{Hz}$).

Next, Figure 4 shows the total system power consumption versus the number of IRS elements *M* in the single-user scenario with the minimum required rate $R_{\mathrm{set}}=12\;\mathrm{bits}/s/\mathrm{Hz}$. Firstly, for the RAND algorithm, the power consumption is the highest of the three algorithms. Secondly, for the NPS algorithm, the power consumption decreases first and then increases. This is because when *M* is relatively small, the beamforming gain provided by a larger IRS helps the BS reduce power consumption. However, when *M* is relatively large, the increased power consumption from the IRS is higher compared with the decreased power consumption from the BS. Furthermore, the power consumption of the 2-bit IRS-aided system is higher than that of the 1-bit IRS-aided system, which shows the necessity of considering the PS-DPC of the multi-bit IRS. Thirdly, the GBD algorithm performs better in the 2-bit IRS scenario than in the 1-bit one, which is more obvious at a smaller *M*. In addition, by comparing the performance of 1-bit and 2-bit IRSs, we can conclude that it is more important to consider the PS-DPC of the IRS and adopt an appropriate algorithm in a multi-bit IRS-aided system than a 1-bit IRS-aided system. Hence, if the IRS has a large size, the beamforming optimization ignoring the PS-DPC may result in high power consumption. If the IRS has a limited size, improving the resolution of the IRS is an effective way to reduce the power consumption.

**Figure 5:**
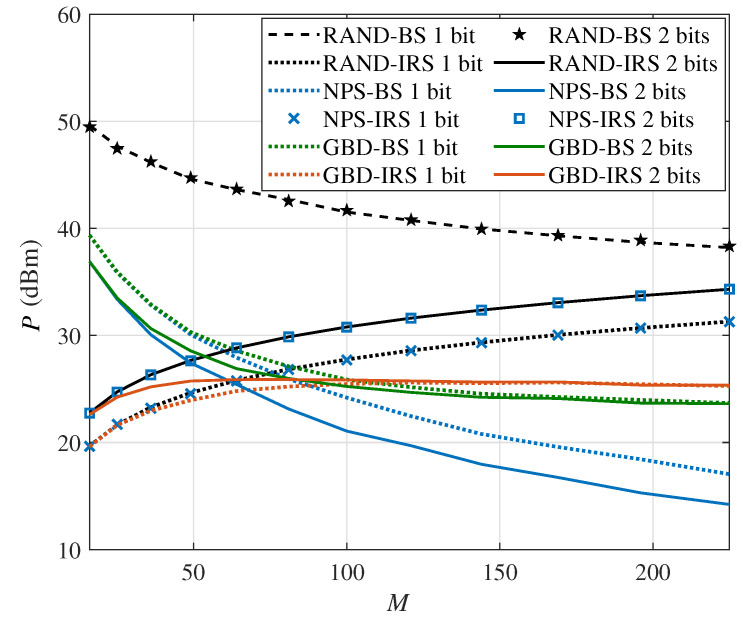
The BS and IRS power consumption versus the number of IRS reflection elements *M* in the single-user scenario ($R_{\mathrm{set}}=12\;\mathrm{bits}/s/\mathrm{Hz}$).

Figure 5 shows the BS and IRS power consumption versus the number of IRS elements *M* in the single-user scenario with the minimum required rate $R_{\mathrm{set}}=12\;\mathrm{bits}/s/\mathrm{Hz}$ in order to further analyze the phenomenon in Figure 4. On the one hand, we analyze the BS power consumption. For the RAND and NPS algorithms, as the larger IRS can provide a higher beamforming gain, the BS power consumption decreases as the number of IRS elements *M* increases. For the GBD algorithm, the trend of the BS power consumption also decreases but is smoother than the RAND and NPS algorithms. On the other hand, we analyze the IRS power consumption. For the RAND and NPS algorithms, as they only consider the beamforming ability of the IRS, the larger IRS has the higher power consumption. Meanwhile, the GBD algorithm keeps the IRS power consumption at a similar level with the BS when the number of IRS elements *M* increases. The difference between the proposed GBD algorithm and other algorithms indicates that the GBD algorithm tends to balance the beamforming pressure, thereby avoiding excessive load on the BS or IRS. In addition, by comparing the performance of 1-bit and 2-bit IRSs in the GBD algorithm, if the IRS has a limited size, a multi-bit IRS can provide more beamforming gain to reduce the BS power consumption.

### 5.3. Multi-User Scenario

In the multi-user scenario, we show the power consumption versus the rate requirement, the number of reflection elements, the resolution of the IRS, and the number of users, thereby analyzing the performance of the RAND algorithm, the NPS algorithm, the CD algorithm, and the GA scheme from these perspectives.

**Figure 6:**
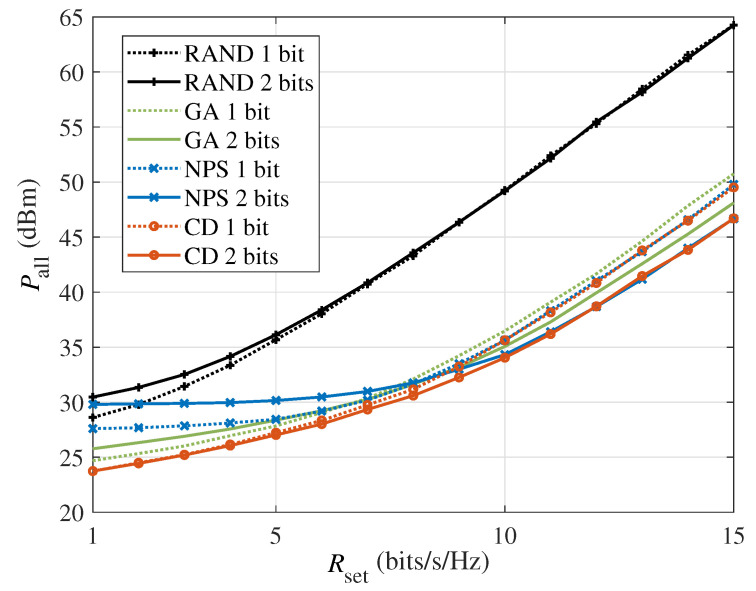
The system power consumption versus the rate requirement $R_{\mathrm{set}}$ in the multi-user scenario (M=64, K=2).

Figure 6 shows the total system power consumption versus the minimum required rate $R_{\mathrm{set}}$ in the multi-user scenario with M=64 and K=2. Compared with Figure 2 of the single-user scenario, since the rate requirement targets multiple users, the power consumption in the multi-user scenario is higher at the same $R_{\mathrm{set}}$. As analyzed previously, if the power consumption of the BS is less than or comparable to the IRS, the proposed CD algorithm has a gain over the NPS algorithm. Hence, in the multi-user scenario, the gap between the CD algorithm and NPS algorithm disappears at a lower required rate $R_{\mathrm{set}}$ than in the single-user scenario. In addition, as an algorithm with lower complexity than the CD algorithm, the GA scheme can also optimize the power consumption of the system.

**Figure 7:**
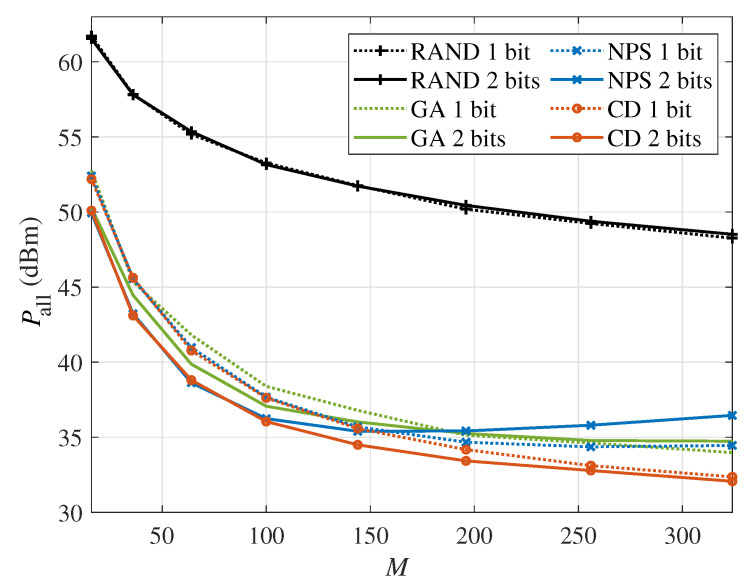
The system power consumption versus the number of IRS reflection elements *M* in the multi-user scenario ($R_{\mathrm{set}}=12\;\mathrm{bits}/s/\mathrm{Hz}$, K=2).

Figure 7 shows the total system power consumption versus the number of IRS elements *M* in the multi-user scenario with the minimum required rate $R_{\mathrm{set}}=12\;\mathrm{bits}/s/\mathrm{Hz}$ and K=2. Compared with Figure 4 of the single-user scenario, the gap between the CD algorithm and NPS algorithm widens at a larger reflection elements number. In addition, the gap appears when the power consumption of the BS is comparable to that of the IRS. Since the total power consumption of the multi-user scenario is higher than in the single-user scenario, the gap appears at a higher level of power consumption for the IRS. Hence, in the multi-user scenario, the gap occurs at a larger number of IRS elements than in the single-user scenario. Therefore, when the IRS has a large number of reflection elements, it is necessary to consider the PS-DPC model for the IRS.

**Figure 8:**
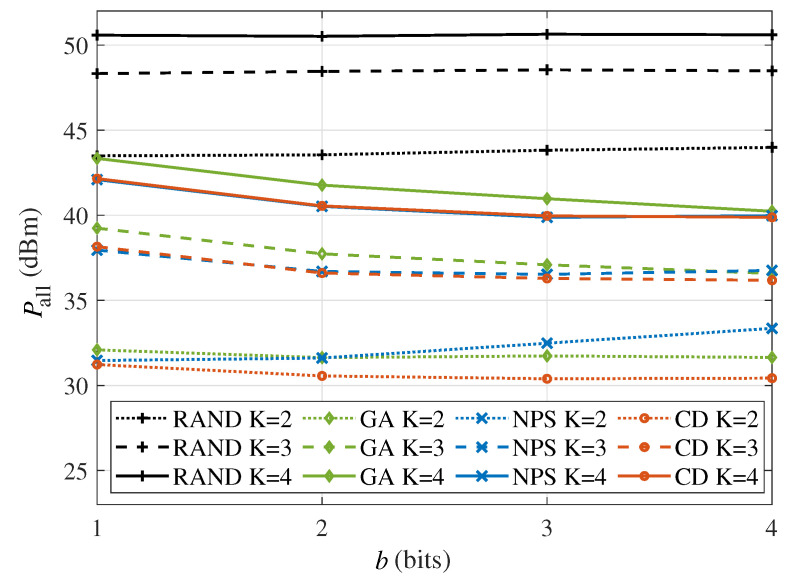
The power consumption versus the phase shift quantization bits *b* in the multi-user scenario ($R_{\mathrm{set}}=8\;\mathrm{bits}/s/\mathrm{Hz}$, M=64).

Figure 8 shows the power consumption versus the phase shift quantization bits, i.e., *b*, for different numbers of users with $R_{\mathrm{set}}=8\;\mathrm{bits}/s/\mathrm{Hz}$ and M=64. On the one hand, the RAND algorithm has the highest power consumption, which remains constant as *b* increases. On the other hand, for the CD algorithm and the GA scheme, the power consumption decreases as the phase shift resolution increases, but it is limited for b≥2. Hence, considering the hardware implementation perspective, the 2-bit or 3-bit IRS is sufficient to meet the requirements in the simulation. In addition, comparing the NPS and CD algorithms, it can be found that the gap between these two algorithms becomes larger when the phase shift quantization bits are larger and the user number is smaller. Furthermore, although the population generation in the GA scheme involves randomness, the retention of beneficial genes and mutations that can introduce new possibilities enables the GA scheme to achieve performance approximately equivalent to the CD algorithm.

**Figure 9:**
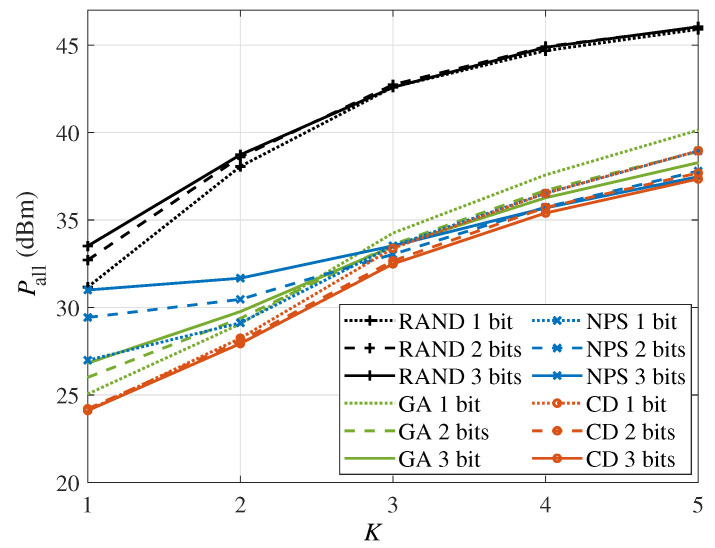
The power consumption versus the number of users *K* in the multi-user scenario ($R_{\mathrm{set}}=6\;\mathrm{bits}/s/\mathrm{Hz}$, M=64).

Finally, Figure 9 shows the power consumption versus the number of users *K* with $R_{\mathrm{set}}=6\;\mathrm{bits}/s/\mathrm{Hz}$ and M=64. It can be seen that the power consumption increases as the number of users increases and slows down when the system has a larger number of users, i.e., K≥4. In addition, comparing the NPS and CD algorithms, it can be found that the gap between these two algorithms becomes smaller as the number of users increases or the IRS resolution decreases. The reason is that in these two scenarios, the total power consumption of the system is high, and the power consumption of the IRS accounts for a small portion of it.

## 6. Conclusions

We have investigated beamforming optimization in multi-bit IRS-aided systems with the practical PS-DPC model. To deal with the implicit and nonlinear relationship between the PS-DPC and the phase shift configuration of the IRS, we introduced a selection matrix to explicitly represent the PS-DPC. Then, we designed the GBD-based beamforming optimization algorithm for the single-user scenario and proposed the CD-based algorithm and a GA-based scheme for the multi-user scenario. The simulation results have verified the effectiveness of our proposed algorithms. On the one hand, if the system requires a lower rate, the IRS has a large number of reflection elements, the IRS has a high reflection resolution, or the system has few users, it is necessary to consider the PS-DPC model of the IRS because an inaccurate power consumption model introduces additional errors and results in high power consumption in practical systems. On the other hand, improving the resolution and increasing the number of elements are both feasible ways to reduce power consumption. Specifically, the effect of improving the resolution performs better at a higher requirement rate and with fewer IRS elements. In addition, the results have also shown that the proposed algorithms can achieve a good trade-off between the transmission data rate and the system power consumption. In future work, the system model can be generalized to more general communication scenarios. The optimization algorithms can also be modified with lower complexity.

## Data Availability

The data are contained within the article.
